# Inverse Design of Thermal Imaging Metalens Achieving 100° Field of View on a 4 × 4 Microbolometer Array

**DOI:** 10.3390/mi17010065

**Published:** 2025-12-31

**Authors:** Munseong Bae, Eunbi Jang, Chanik Kang, Haejun Chung

**Affiliations:** 1 Department of Electronic Engineering, Hanyang University, Seoul 04763, Republic of Korea; munseong97@hanyang.ac.kr; 2 Department of Artificial Intelligence Semiconductor Engineering, Hanyang University, Seoul 04763, Republic of Korea; rloldl@hanyang.ac.kr; 3 Department of Artificial Intelligence, Hanyang University, Seoul 04763, Republic of Korea; chanik@hanyang.ac.kr

**Keywords:** metalens, inverse design, adjoint optimization, wide field of view (FoV), long-wave infrared (LWIR), thermal imaging, low-resolution IoT sensors

## Abstract

We present an inverse designed metalens for long-wave infrared (LWIR) imaging tailored to consumer and Internet of Things (IoT) platforms. Conventional LWIR optics either rely on costly specialty materials or suffer from low efficiency and narrow fields of view (FoV), limiting scalability. Our approach integrates adjoint-based inverse design with fabrication-aware constraints and a cone-shaped source model that efficiently captures oblique incidence during optimization. The resulting multi-level metalens achieves a wide FoV up to 100° while maintaining robust focusing efficiency and stable angle-to-position mapping on low-power 4×4 microbolometer arrays representative of edge devices. We further demonstrate application-level imaging on 4×4 microbolometer arrays, showing that the proposed metalens delivers a substantially wider FoV than a commercial narrow FoV lens while meeting low-resolution, low-cost, and low-power constraints for edge LWIR modules. By eliminating bulky multi-element stacks and reducing cost and form factor, the proposed design provides a practical pathway to compact, energy-efficient LWIR modules for consumer applications such as occupancy analytics, smart-building automation, mobile sensing, and outdoor fire surveillance.

## 1. Introduction

Long-wave infrared (LWIR; 8–12 μm ) sensing has emerged as a critical technology in consumer electronics and Internet of Things (IoT) applications over the past decade [1,2,3,4,5]. Beyond traditional tasks such as footfall counting [6] and indoor search-and-rescue [7], many contemporary systems leverage LWIR for non-contact thermometry [8,9], motion and presence detection [10,11], occupancy-aware HVAC control [12,13], crowd-flow analytics [14,15], driver-assistance under reduced-visibility conditions [16,17], and mobile perception [18]. These functions are increasingly embedded in smart buildings [19,20,21], security infrastructures [22,23], autonomous and semi-autonomous vehicles [24], and handheld devices [25,26,27]. Continued cost reductions, advances in micro-fabrication [28,29] and readout circuitry [30,31,32], and the integration of edge-AI inference [33,34,35] have, in turn, established low-cost, uncooled microbolometer arrays [36] as the predominant detector technology for consumer-grade LWIR imaging [37,38].

Despite growing demand, current LWIR sensing systems often exhibit a limited field of view (FoV) [39,40,41]. A principal cause is the behavior of Fresnel optics at high numerical aperture (NA): as NA increases, groove shadowing and vignetting, profile quantization, and off-axis diffraction–efficiency roll-off reduce throughput and degrade performance at large oblique incidence [42]. For example, a 4×4mm Fresnel aperture with focal length f=3.5mm provides an FoV of ≈60° with insufficient irradiance at the sensor; shortening the focal length to f=1.43mm increases the FoV to ≈109° but further diminishes collected energy due to suboptimal transmission and poor grating efficiency at high incident angles. At the other end of the design space, relaxing FoV constraints typically requires bulky optics with longer back focal distances and/or expensive, high-resolution LWIR focal-plane arrays [43], which increases both package size and cost.

To enable low-cost, low-power LWIR applications, such as footfall analytics and indoor/outdoor fire detection [44,45,46], we propose a wide-FoV silicon metalens paired with a low-power, low-cost 4×4 uncooled LWIR microbolometer array [47,48].

**Figure 1:**
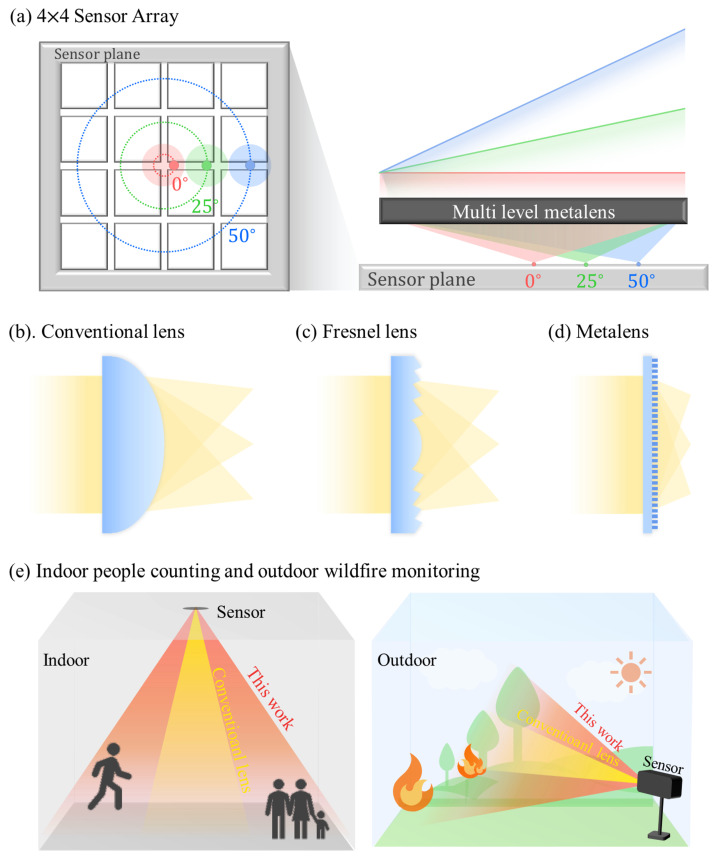
(**a**) Proposed multi-level thermal lens array for wide-angle infrared imaging. The designed structure enables linear focusing at multiple incident angles (0°, ±25°, ±50°) onto a sensor plane. (**b**) Conventional convex lens, (**c**) Fresnel lens, (**d**) metalens, and (**e**) conceptual applications including indoor people counting and outdoor wildfire monitoring, demonstrating the extended FoV of the proposed lens compared to conventional optics.

Our proposed architecture is summarized in Figure 1. As illustrated in Figure 1a, the approach achieves linear angle-to-focus mapping at 0°, ±25°, and ±50° across a 4×4 sensor array. This stands in contrast to conventional bulky refractive optics, as shown in Figure 1b, and to Fresnel lenses, as shown in Figure 1c, which are typically FoV-limited and poorly suited for compact or low-power systems [49]. In contrast, the metalens offers a thin and scalable metasurface architecture, as shown in Figure 1d. The resulting wide-FoV extends functionality to consumer/IoT scenarios, as shown in Figure 1e, enabling single-frame scene capture at fixed sensor power and array size. These results collectively motivate a silicon-based flat-optics approach.

Metalenses employ subwavelength nanostructures to impose spatially varying phase, amplitude, and polarization on the optical field, enabling lightweight form factors, straightforward on-chip integration, and precise wavefront control for focusing, illumination homogenization, and beam steering [50,51,52,53,54,55].

Beyond consumer and IoT applications, metalenses have also attracted growing interest in bioimaging and biomedical sensing, where compact form factors and integration-friendly architectures are advantageous for point-of-care diagnostics, wearable monitoring, and endoscopic modalities [56]. Although this work focuses on LWIR thermal sensing, the proposed wide-FoV optimization framework and fabrication-aware constraints may provide useful design insights for related flat-optics platforms in adjacent application domains.

Silicon is transparent across the LWIR band and exhibits a high refractive index ( n≈3.4 ), allowing strong phase control within a compact volume; thin planar Si metasurfaces can therefore efficiently redirect and focus LWIR radiation while remaining compatible with monolithic sensor integration [57]. A key remaining challenge, however, arises at high NA: library-driven meta-atom designs must realize rapidly varying phase and amplitude across the aperture, which increases spatial-frequency demands and leads to efficiency roll-off, aberrations, and sensitivity to oblique incidence [58,59]. The local periodic approximation underlying unit-cell libraries neglects inter-element coupling and degrades off-axis performance—often manifesting as 9–22% focal shifts and reduced efficiency [60,61].

Inverse design has emerged as a powerful approach for optimizing complex photonic functionalities with high efficiency [62,63]. Among these techniques, adjoint-based topology optimization enables the evaluation of the gradient of a chosen figure of merit with respect to extremely large numbers of design variables using only two full-wave simulations: a forward solve and an adjoint solve [64,65]. This approach effectively renders the cost of gradient computation independent of the parameter count. This computational efficiency, in turn, enables large-scale optimization and practical realization of multifunctional metasurfaces [66,67,68,69].

In this work, we introduce a low-cost, low-power, and ultra-compact wide-FoV thermal imaging architecture aimed at footfall analytics and indoor/outdoor fire detection. To realize ultra-compact, wide-angle operation, we design a multi-level LWIR silicon metalens using adjoint-based topology optimization tailored to grayscale lithography [70,71]. In contrast to high-resolution imagers that trade power and cost for fidelity, our approach targets resource-constrained IoT sensor arrays while preserving wide-FoV performance up to ±50° incidence. The metalens is optimized over the 4mm aperture via inverse design with adjoint gradients under fabrication-aware constraints, including height quantization and a specified minimum feature size. In addition, we develop a multi-angle source modeling strategy that captures oblique-incidence physics without the exponential computational burden of explicit mode summation. The resulting angle-multiplexed, intentionally out-of-focus mapping yields an approximately linear focal displacement over wide angles using a single planar element, enabling substantial gains in spatial mapping and people counting with 4×4 LWIR microbolometer arrays and offering a practical route toward scalable, low-cost manufacturing.

## 2. Methodology

In this section, we introduce the overall methodology and demonstrate the design of a large-scale, wide-FoV LWIR metalens. The target device is a 3.5μm -thick $4\times 4\;{\mathrm{mm}}^{2}$ cylindrical metalens on a 400μm thick silicon substrate. The focal length was set to $f_{\mathrm{eff}}=1.667\;\mathrm{mm}$ (corresponding to NA≈0.77 ) for the integration between the metalens and the sensor. In more detail, single-layer anti-reflection (AR) coatings ( n≈1.86 ) on both surfaces with thicknesses of 2μm and 3μm are placed to suppress Fresnel reflections at the design wavelength of λ=8μm . For electromagnetic modeling, three optical media were defined: air ( n=1.0 ), crystalline silicon ( n=3.4698 ), and the AR coating ( n=1.8627 ), which were used consistently in the optimization process.

From this perspective, we first introduce the concept of a cone-shaped source to efficiently realize horizontal and vertical FoV across our inverse design framework using full-wave simulations. Subsequently, we introduce an adjoint compatible fabrication-aware algorithm for low-cost manufacturing.

At the end of this section, we take a sequential approach to realizing a low-cost 4mm ( 500λ ) target device with wide-FoV by demonstrating the above concepts on a 0.2mm ( 25λ ) aperture metalens with 100° FoV and fabrication feasibility.

### 2.1. Cone-Shaped Source for the Wide-FoV Configuration

The aperture size 4mm ( 500λ ) requires massive voxels (>20,000) along the horizontal axes for accurate simulation. This requirement is squared under the three-dimensional Cartesian coordinates and exponential to the computational cost for the simulation.

To reduce this cost, we employed a 2.5-dimensional (2.5D) simulation framework with cylindrical symmetry. In this framework, the angular dependence of the continuous fields can always be chosen in the form $e^{im\phi }$ for a certain integer *m*:

(1) $$E_{m}\left(\rho ,z\right)=E\left(\rho ,0,z\right)e^{im\phi },$$

where $E_{m}\left(\rho ,z\right)$ is a field of ρz plane with angular dependence mϕ . This is essentially a 1D calculation only for the ρ direction. For this reason, the simulation is even faster than the conventional 2D calculation [72]. Specifically, when the integer number *m* is −1, a normal incidence field with left-handed circular polarization (LCP) can be implemented with a single 2.5D simulation:

(2) $$E_{\mathrm{LCP}}\left(\rho ,\phi ,z\right)=E_{-1}\left(\rho ,z\right)\hat{\rho }+iE_{-1}\left(\rho ,z\right)\hat{\phi }.$$

**Figure 2:**
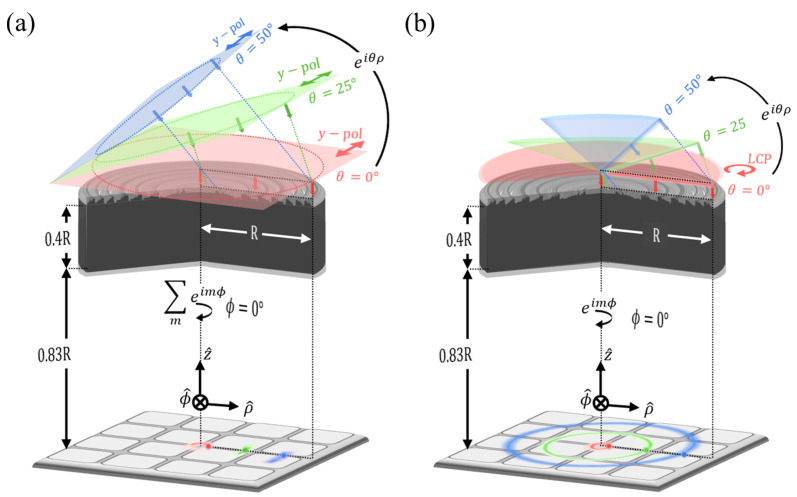
Modeling of oblique incidence within cylindrical symmetry. Each source is specified with elevation angles ( $\theta =0^{{}^{\circ}}$ (red), $\theta =25^{{}^{\circ}}$ (green), $\theta =50^{{}^{\circ}}$ (blue)). Corresponding focused field profiles are projected on the sensor plane. The focal length between the bottom of the lens and the sensor plane is 0.8 times the lens radius R. (**a**) Reconstructed oblique planewave incidents from superposition of cylindrical harmonics exp(imϕ) ( m=−N,…,N ), enabling accurate reconstruction of linearly polarized oblique incidence. (**b**) Cone-shaped source incidents from a single exp(imϕ) ( m=−1 ), enabling efficient configuration of circularly polarized oblique incidence for cylindrical lens geometry.

However, unlike the normal incidence, a single 2.5D simulation cannot implement oblique incidence. The oblique planewave can be defined as a superposition of the decomposed planewave components across a wide range of *m* from multiple 2.5D simulations for each *m* as shown in Figure 2a [73].

To squeeze these into a single 2.5D simulation for efficient consideration of the oblique incidence, we defined the cone-shaped source with the angular dependence of mϕ as follows:

(3) $$E_{\theta }\left(\rho ,\phi ,z\right)=\left[E_{-1}\left(\rho ,z\right)\hat{\rho }+iE_{-1}\left(\rho ,z\right)\hat{\phi }\right]e^{i2\pi \lambda^{-1}\sin\theta \;z},$$

where θ is the elevation angle of the oblique incidence.

Figure 2b illustrates an overview of our lens problem under the cone-shaped source for three sampling angles ( θ=0°,25° , and 50°), where *m* is −1 to represent LCP in the ρz plane.

Since geometry and fields have cylindrical symmetry, each simulation considers the response for all azimuthal directions at a given elevation angle. Specifically, when θ=0° , a cone-shaped source is equivalent to a normal incidence of a planewave with LCP, leading to polarization insensitivity for the normal incidence. On the other hand, when θ≠0° , a cone-shaped source cannot illuminate the center area of the lens (as shown as dashed green and blue lines in Figure 2b), and this can lead to polarization sensitivity.

We define the wide-FoV thermal imaging problem as a multi-objective optimization of far fields at the sensor area. Specifically, our optimization problem considers the focusing of the three cone-shaped sources ( θ=0° , θ=25° , and θ=50° ) into linearly spaced target focal points throughout the sensor area in the ϕ=0° plane. The figure of merit of our optimization problem is defined as

(4) $$\mathrm{FoM}=|E_{0{}^{\circ}}\left(\rho_{0},z_{s}\right){|}^{2}+|E_{25{}^{\circ}}\left(\rho_{1},z_{s}\right){|}^{2}+{\left|E_{50{}^{\circ}}\left(\rho_{2},z_{s}\right)\right|}^{2},$$

where $E_{\theta }$ denotes the approximated far-field at the focal point from the near-field response of the geometry and cone-shaped sources with θ , and $\left(\rho_{0,1,2},z_{0}\right)$ are linearly spaced focal points along the sensor area. The near-to-far approximation can reduce the computational cost for wave propagation between the geometry and the sensor area within the iterative optimization processes.

### 2.2. Adjoint Compatible Fabrication-Aware Algorithm

We formulate the metalens design as an adjoint-optimization inverse problem. Rather than prescribing a local phase profile [74] or assembling a unit-cell library [75], the aperture is parameterized as a spatially varying height (or effective permittivity) field that is iteratively updated to maximize a FoM. The FoM aggregates focusing performance over a set of sampled incidence angles to realize a wide field of view, evaluating light concentration within prescribed focal windows on the sensor plane.

**Figure 3:**
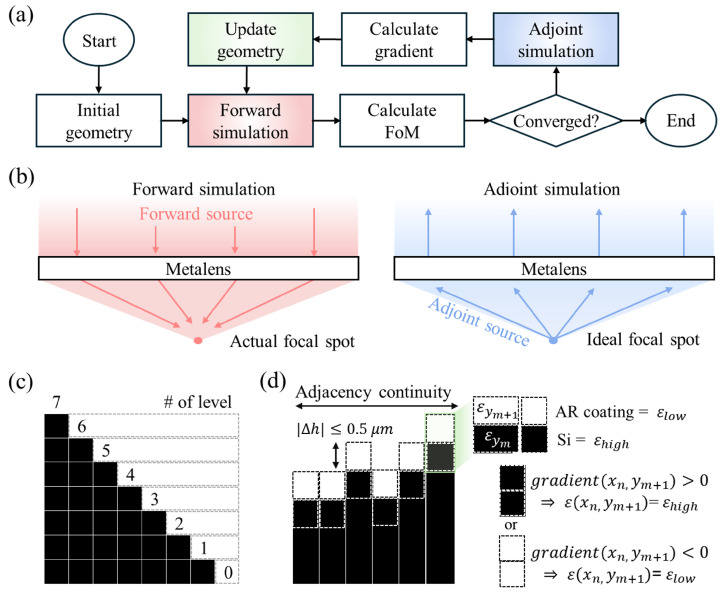
Adjoint-optimization workflow and fabrication-aware constraints. (**a**) An iterative optimization loop begins with an initial geometry: a forward full-wave simulation with FoM evaluation is followed by an adjoint simulation for gradient computation; the geometry is updated until convergence. (**b**) Schematics of forward and adjoint simulations. In the forward simulation, the source generates the actual focal spot; in the adjoint simulation, a source is placed at the target focal spot and propagated backward to compute adjoint sensitivities. (**c**) Local material update at the adjoint-defined boundary: if the gradient at cell $\left(x_{n},y_{m+1}\right)$ is positive (negative), the permittivity is driven toward $\varepsilon_{\mathrm{high}}$ ( $\varepsilon_{\mathrm{low}}$ ). (**d**) Constraints enforced each iteration: (i) step of design-region pixel; (ii) adjacency continuity to maintain a connectivity; and (iii) bounded inter-cell height step, Δh≤0.5μm .

Figure 3a summarizes the optimization loop as an adjoint method-based optimization cycle. As illustrated in Figure 3b, the forward step performs a full-wave solve for each sampled angle to compute the device response and contribute to the multi-angle FoM. In the adjoint step for the same angle, a reciprocal source is placed at the target focal spot on the sensor plane and propagated backward through the structure. Consequently, one forward and one adjoint simulation per angle provide gradients with respect to all design variables, effectively decoupling the cost of gradient evaluation from the number of parameters. Fabrication-aware constraints, which are described in Figure 3c, are enforced at each iteration. The optimization loop is terminated when the multi-angle FoM converges over several successive iterations.

### 2.3. Demonstrations on Small-Scale Metalens

To realize the target device, we take demonstrations of our concepts in the prior subsections on small-scale metalens design. The focusing efficiency of metalens is evaluated with field intensity within the 1.75μm range of the focal monitor centered at each focal points.

**Figure 4:**
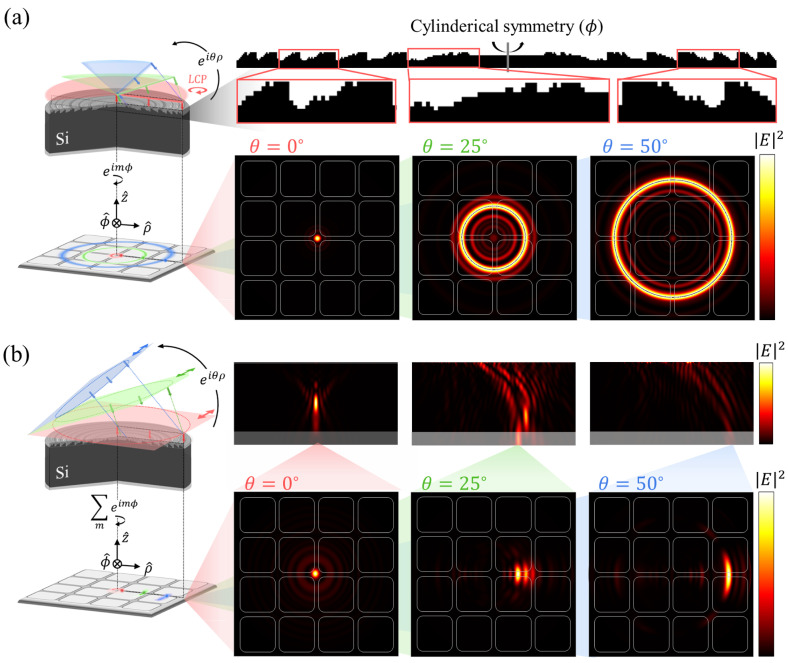
Demonstration of the inverse designed 0.2mm lens. (**a**) Optimized geometry and demonstration under the cone-shaped sources. The optimized lens geometry has an aperture size of 0.2mm on 0.04mm thick Si substrate. The focused field profiles on the sensor plane under the LCP cone-shaped sources are highlighted with elevation angles ( $\theta =0^{{}^{\circ}}$ (red), $\theta =25^{{}^{\circ}}$ (green), $\theta =50^{{}^{\circ}}$ (blue)). (**b**) Demonstration under the linearly polarized oblique incidence. The oblique planewave incidents reconstructed from the superposition of 201 cylindrical harmonics exp(imϕ) ( m=−100,…,100 ). The focused field profiles on the sensor plane under the y-polarized oblique planewaves are highlighted with elevation angles ( $\theta =0^{{}^{\circ}}$ (red), $\theta =25^{{}^{\circ}}$ (green), $\theta =50^{{}^{\circ}}$ (blue)). The corresponding vertical intensity profiles are shown in the top subplots.

Figure 4 shows the demonstration of an inverse designed 0.2mm lens under the LCP cone-shaped sources and y-polarized oblique planewaves. The evaluations of LCP and linear polarized incidence can be considered the focusing efficiency under the unpolarized incidence from thermal radiation. In greater detail, Figure 4a shows optimized 0.2mm lens geometry and focused field intensity from the cone-shaped source. The focusing efficiency under the LCP cone-shaped sources ( θ=0°,25°, and 50° ) are 11.6%, 19.4%, and 14.7%. Subsequently, Figure 4b depicts propagating field intensity within the vertical cross-section, and focused field intensity from the y-polarized oblique planewave within the horizontal sensor plane. The focusing efficiency under the y-pol oblique incidences are 11.5%, 22.1%, and 14.2%.

The Root Mean Square Error (RMSE) between both demonstrations is 1.586%, indicating that a cone-shaped source can be considered polarization-insensitive for oblique incidence with cylindrical geometry.

The observed polarization-insensitive behavior can be attributed to the cylindrical symmetry of the proposed metalens. Because the structure is rotationally invariant about the optical axis, an incident linear polarization can be expressed as two orthogonal components whose responses become equivalent after azimuthal averaging. Accordingly, the LCP response obtained from a single m=−1 simulation serves as a computationally efficient proxy for unpolarized thermal radiation, which is the relevant source condition for LWIR imaging. Similar symmetry-based simplifications have been adopted in prior cylindrically symmetric metalens studies [76].

## 3. Results

In this section, we demonstrate a 4mm aperture metalens by validating it against a small-scale 0.2mm aperture metalens from the previous section and presenting quantitative results on wide-angle behavior and focusing efficiency over the 35μm range of the focal monitor. Since the fidelity to cone-shaped sources was validated on the small-scale metalens (see Figure 4), we demonstrate only circularly polarized cone-shaped sources at the application scale, given the computational constraints.

**Figure 5:**
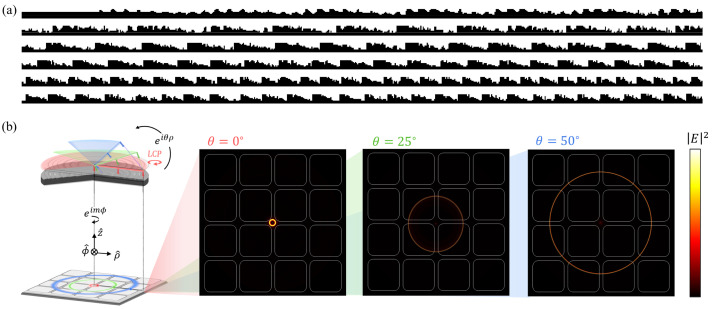
Demonstration of the inverse designed 4mm metalens. (**a**) Optimized multi-level geometry under fabrication-aware constraints. The 4mm aperture lens is implemented on a 0.4mm thick silicon substrate with eight quantized height levels. (**b**) Far-field focusing characteristics under cone-shaped sources at elevation angles $\theta =0^{{}^{\circ}}$ (red), $\theta =25^{{}^{\circ}}$ (green), and $\theta =50^{{}^{\circ}}$ (blue). The left schematic illustrates the cone-shaped source illumination with LCP and its angle-dependent mapping onto the 4×4 sensor plane. The corresponding sensor plane intensity maps confirm linear focal displacement with increasing incidence angle, while maintaining consistent focal confinement. Color bar represents normalized field intensity ${\left|E\right|}^{2}$ .

Figure 5a shows the optimized 4mm aperture metalens obtained under the fabrication-aware, eight-level surface-relief parameterization on a 0.4mm Si substrate. Due to the cylindrical symmetry, the 2mm radius was partitioned into 4000 concentric cells. A bit-plane encoding with seven binary layers enforces eight quantized height levels, yielding 4000 × 7 = 28,000 design variables.

Figure 5b reports focused field intensity under LCP cone-shaped sources at θ=0° , 25° , and 50° . The corresponding focusing efficiencies are 14.6% , 15.5% , and 12.7% , respectively. Specifically, the 50° case retains ∼87% of the normal-incidence throughput.

While the absolute focusing efficiencies (12–15%) are lower than those of diffraction-limited on-axis metalenses, the target use case here is low-resolution thermal sensing rather than high-fidelity imaging. For 4×4 microbolometer arrays used in occupancy detection, fire surveillance, and presence sensing, a key requirement is stable and angle-consistent energy delivery within each pixel area across the FoV, because contrast-based detection relies primarily on relative signal differences rather than fine spatial detail. In this regard, the proposed metalens maintains comparable efficiency across incidence angles ( 12.7 – 15.5% ), which supports consistent pixel-level throughput over the wide FoV. Moreover, uncooled microbolometers are commonly reported to exhibit Noise Equivalent Temperature Difference (NETD) values on the order of tens of millikelvin [77], suggesting that detection performance is typically dominated by scene-level thermal contrast and system-level calibration rather than solely by maximizing optical throughput.

This angle-uniformity is consistent with the multi-objective formulation in (4) that maximizes the summation of intensities at three prescribed focal regions on the sensor plane. Consequently, our design produces lateral focal displacement for angle-to-position mapping on the 4×4 array sensor for thermal imaging applications.

This functionality is advantageous for low-power, low-resolution thermal modules in consumer and IoT platforms: rather than requiring diffraction-limited on-axis focusing, the system exploits angle-stable, wide-FoV collection and reliable spot registration across the array, thereby simplifying downstream detection while maintaining scene coverage.

We quantified the imaging performance of the designed metalens using a point-spread function (PSF)–modulation transfer function (MTF) framework [78]. Full-wave electromagnetic simulations were carried out at incidence angles of 0°, 25°, and 50° to obtain sensor plane field distributions. From these data, the sensor plane PSF I(ρ,ϕ) was extracted for each angle, azimuthally averaged to yield a radial PSF I(r) , and reconstructed into an axisymmetric two-dimensional PSF I(x,y) via concentric-ring interpolation. Each PSF was normalized such that its total integrated intensity equals unity, which guarantees that the optical transfer function (OTF), obtained via Fourier transformation, satisfies OTF(0)=1 . The MTF was then computed as |OTF| and azimuthally averaged to give the radial MTF M(ν) , where the spatial frequency *f* was normalized by the incoherent diffraction-limited cutoff $f_{c}=2\;\mathrm{NA}/\lambda$ so that $\nu =f/f_{c}$ . All MTFs are reported alongside the theoretical incoherent diffraction-limited MTF for quantitative comparison.

**Figure 6:**
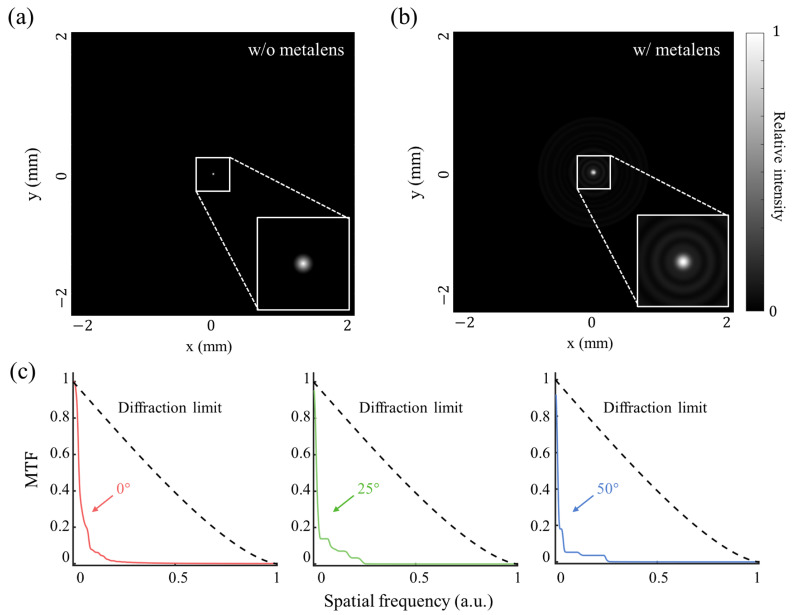
PSF-MTF analysis of the 4mm metalens at the design wavelength under the LCP cone-shaped source model for oblique incidence. (**a**) Sensor plane intensity without the metalens at normal incidence, showing a near-diffraction-limited focal spot with minimal sidelobes. (**b**) Sensor plane intensity with the metalens at normal incidence; the focus remains comparably confined, while weak concentric Airy-like rings appear due to residual phase quantization and fabrication-constrained profile discretization. (**c**) Azimuthally averaged MTFs for incidence angles of 0° (red), 25° (green), and 50° (blue), computed from PSFs normalized to unit energy and plotted relative to the incoherent diffraction-limited MTF (black dashed).

Figure 6 presents the results of the PSF-MTF analysis. Figure 6a,b show representative on-axis ( θ=0° ) PSFs for the reference system without the metalens and for the proposed metalens. The reference PSF in Figure 6a exhibits a clean diffraction-limited spot, whereas the metalens PSF in Figure 6b retains a confined main lobe accompanied by weak concentric Airy-like rings. These residual rings are consistent with phase quantization and profile discretization under the eight-level constraint and indicate a small but measurable departure from the ideal wavefront. Figure 6c compares angle-dependent MTFs computed from PSFs at θ=0° (red), 25° (green), and 50° (blue). At low spatial frequencies, all three curves remain close to the incoherent diffraction limit, indicating stable transfer of large-scale structures and edges. In contrast, the mid- to high-frequency response decays more rapidly, leading to reduced contrast for fine details and textures. This roll-off steepens with increasing field angle, yielding more pronounced peripheral contrast loss toward 20°.

These results indicate an inherent trade-off between FoV expansion and spatial resolution in wide-angle metalens design. Achieving a 100° FoV with a single planar element tends to introduce increased off-axis aberrations and diffraction effects at large incidence angles, which manifest as a reduction in mid- and high-spatial-frequency contrast. However, for the targeted low-resolution IoT applications, the spatial-frequency content of interest lies primarily in the low-to-mid frequency range, where the MTF remains close to the incoherent diffraction limit across all field angles. Accordingly, since presence detection and thermal anomaly identification rely on scene-level contrast rather than fine spatial detail, the observed high-frequency MTF degradation does not limit the practical applicability of the proposed metalens.

Meanwhile, because the cutoff frequency set by the given NA and wavelength is identical for all angles, the MTF preserves near diffraction-limited performance on-axis while exhibiting diminished mid/high-frequency contrast at larger angles. Nevertheless, the effective passband remains comparable across the angle range, supporting consistent scene rendition under wide-FoV conditions.

To approximate application-level behavior over a wide field, we formed a composite MTF by equally averaging the angle-specific MTFs. We used it as a radial OTF mask, |OTF(ν)|=M(ν) , for computational reconstructions of real thermal scenes. Consistent with the angle-dependent trends in Figure 6c, this composite response preserves low spatial frequencies well but attenuates mid-to-higher frequencies more strongly, so reconstructed images retain scene structure and edge contrast while very fine textures appear softened.

**Figure 7:**
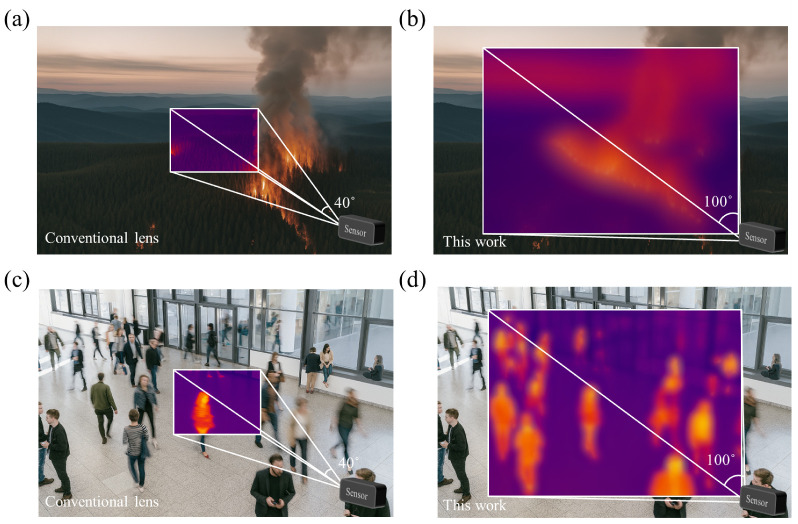
Application-level comparison between a conventional narrow-FoV lens and the proposed wide-FoV metalens under identical scene and sensor conditions. (**a**) Conventional lens with a 40° FoV capturing only a limited part of the wildfire thermal scene. (**b**) Proposed metalens with a 100° FoV providing single-frame coverage of the full wildfire extent. (**c**) A conventional narrow FoV lens records only a localized region, constraining people counting/occupancy estimation. (**d**) The wide-FoV metalens yields scene-level coverage in a single frame, enabling reliable human activity recognition and people counting.

Figure 7 compares narrow-FoV (40°) and wide-FoV (100°) cases under identical sensor conditions. The conventional optic [Figure 7a,c] captures only a localized region, limiting wildfire monitoring and people counting, whereas the proposed metalens [Figure 7b,d] delivers scene-level coverage in a single frame with task-relevant contrast. In line with Figure 6c, the wide-FoV reconstructions exhibit modest high-frequency roll-off (perceived as slight blur in fine textures), but maintain separability of salient features and boundaries across the field. These results indicate that the metalens provides low-power, low-cost LWIR modules in IoT platforms (e.g., occupancy analytics, smart-building automation, and outdoor fire surveillance) without multi-element stacks or mechanical scanning.

## 4. Discussion and Conclusions

This work integrates fabrication-aware constraints into an adjoint-based inverse-design framework for a 4mm aperture, demonstrating that a single planar LWIR metalens can provide wide-FoV imaging on resource-constrained sensor arrays and thereby address long-standing challenges in consumer and IoT applications.

At a 0.2mm aperture, we quantitatively validated a cone-shaped source model against a cylindrical-harmonic plane-wave expansion, showing close agreement in focusing efficiency and establishing the model as an efficient computational surrogate for oblique incidence. At the full 4mm aperture, the metalens maintains robust focusing up to ±50°, preserving ∼87% of the normal-incidence throughput at 50° despite practical manufacturing limits (eight height levels and a specified minimum feature size).

The fabrication-aware constraints in this work were selected to be compatible with commonly used silicon micro-fabrication processes. The eight-level height quantization can be implemented, for example, via multi-mask grayscale lithography or sequential deep reactive-ion etching (DRIE) steps, which have been widely employed for micrometer-scale silicon patterning [79]. The minimum feature-size constraint ( 0.5μm ) and the bounded inter-cell height step ( Δh≤0.5μm ) encourage gradual height transitions, which may reduce sensitivity to etch-lag and profile rounding compared with abrupt, high-aspect-ratio features. A full robustness analysis under explicit fabrication tolerances (e.g., etch-depth and linewidth variations) is beyond the scope of this simulation study and is left for future experimental validation.

From a system perspective, the multi-objective FoM yields angle-dependent focal mapping across a 4×4 array, enabling single-frame scene capture and overcoming the localized coverage inherent to commercial narrow-FoV lenses. A PSF–MTF analysis indicates that, across all field angles, the low spatial-frequency response remains near the incoherent diffraction limit—preserving large-scale structures and edges—while mid/high frequencies show a progressive roll-off with increasing angle. The overall attenuation profiles are similar across angles, reflecting uniform behavior over the wide FoV and resulting in only mild high-frequency blur, suitable for low-power, task-oriented LWIR imaging.

Looking ahead, three extensions are most impactful: (i) incorporating a multiwavelength, joint objective with spectral weighting over the 8– 12μm band to improve robustness to device- and scene-level spectral variation; (ii) reformulating the figure of merit to partially redirect oblique incidence toward the optical axis (e.g., telecentric/angle-to-axis remapping) to increase on-axis efficiency for general-purpose imaging; and (iii) translating the fabrication-aware constraints used during optimization (discrete height quantization, sidewall taper, minimum line widths, material absorption) into prototype fabrication and application-relevant testing.

In summary, combining a computationally efficient multi-angle source model, fabrication-aware constraints, and adjoint optimization over the 4mm aperture realizes wide-FoV, high-efficiency LWIR imaging in a single planar element. This establishes a practical design-to-manufacturing pathway for scene-level coverage on low-resolution arrays under strict size, power, and cost budgets typical of consumer/IoT platforms, while providing a foundation for broadband extensions and experimental validation toward scalable, application-specific metalenses.

## Data Availability

All data used in this study will be made available upon reasonable request to the corresponding authors.

## Abbreviations

The following abbreviations are used in this manuscript:

LWIR	Long-wave infrared
FoV	Field of View
IoT	Internet of Things
NA	Numerical Aperture
LCP	Left-handed Circular Polarization
RMSE	Root Mean Square Error
PSF	Point-Spread Function
MTF	Modulation Transfer Function
OTF	Optical Transfer Function
HVAC	Heating, Ventilation, and Air Conditioning
